# Nd: YVO_4_ Laser versus Hydrogen Peroxide for Glass Fiber Post Conditioning: Comparative Analysis of Bond Strength and Surface Roughness with Self-Adhesive and Conventional Dual-Cure Resin Cements

**DOI:** 10.12669/pjms.42.6.15983

**Published:** 2026-06

**Authors:** Dania El Azzouni

**Affiliations:** Dania El Azzouni, Department of Oral and Maxillofacial Prosthodontics, Faculty of Dentistry, King Abdulaziz University, Jeddah, Kingdom of Saudi Arabia

**Keywords:** Conventional dual cure resin cement, Glass fiber posts, Hydrogen peroxide, Neodymium-doped yttrium orthovanadate, Self-adhesive dual cure resin cement

## Abstract

**Objectives:**

To evaluate the effects of various surface conditioners, hydrogen peroxide (H_2_O_2_+Silane) and neodymium-doped yttrium orthovanadate (Nd: YVO_4_ laser + Silane) on the push out bond strength (PBS) and surface roughness (R_a_)of glass fiber post (GFPs) bonded to root dentin using self-adhesive dual cure resin cement (SADCRC) and conventional dual cure resin cement (CDCRC).

**Methodology:**

The present in vitro study was approved by King Abdulaziz University and lasted four months, from 1^st^ July 2025 to 30^th^ October 2025. Sixty single-rooted premolars were decoronated. Root canal preparation was initiated, followed by mechanical preparation and obturation. Post space was prepared using a Peeso reamer. Seventy-five GFPs were split into three groups based on the conditioning regime (n=25): Group-1, No conditioning, Group-2 (H_2_O_2_+Silane), and Group-3 (Nd: YVO4 laser + silane). The profilometer measured R_a_ on five GFP. Based on the type of resin cement used, 20 GFPs from each group were again divided into subgroups A and B. PBS was assessed using a universal testing machine, and failure mode analysis was performed using a stereomicroscope. *ANOVA* and *post hoc Tukey* test were used to identify statistically significant differences (p<0.05).

**Results:**

The highest R_a_ (1.32 ± 0.22 µm) was observed in Group-2 (Nd: YVO_4_ laser) samples. In contrast, Group-1 (Control) had the lowest Ra (*0.43 ± 0.11* µm). The cervical third of Group-3A (Nd: YVO4 laser-silane + CDCRC) (11.26±1.17 MPa) displayed the highest bond strength outcome. Whereas, the apical third of Group-1A (No conditioning- Silane+CDCRC) (3.78±0.21 MPa) exhibited the lowest bond strength.

**Conclusion:**

A neodymium-doped yttrium orthovanadate laser can be a suitable alternative to Hydrogen peroxide. Self-adhesive resin cement exhibited satisfactory performance in all three thirds of the canal.

## INTRODCUTION

Glass fiber posts (GFPs) are widely used in endodontically treated teeth when reinforcement of core build-up materials and support for subsequent indirect restorations are required.^1^ Owing to their lower modulus of elasticity compared with metal posts, GFPs facilitate a more uniform distribution of functional stresses along the post–dentin complex.^2^ Restorations employing GFPs demonstrate superior long-term durability, with effective adhesion between the post, resin cement, and dentin being a critical determinant of clinical success.^1^,^2^

Past evidence reported that applying a silane coupling agent to the post surface can chemically enhance the bond strength with resin cement, without the need for prior surface treatment.^3^ Nevertheless, the effectiveness of silanization in increasing the retention of glass fiber posts remains controversial, prompting investigation into other surface treatment approaches.^4^ Hydrogen peroxide (H_2_O_2_) is generally considered the conventional method in dentistry for enhancing the surface roughness (R_a_) of GFPs, thereby increasing the mechanical retention of the bonding cement. However, the aggressive nature of this chemical agent may damage the surrounding tooth structure.^5^

Various lasers have been used due to their advantages in many areas of dentistry.^6^,^7^ Many researchers have recently become interested in the Neodymium-doped Yttrium Orthovanadate (Nd: YVO_4_) laser due to its remarkable mechanical and physical properties.^8^ A short pulse width and high-power density characterize this laser. According to AlRefeai *et al.*, the PBS of GFPs to resin cement may be enhanced by using an Nd: YVO_4_ laser as a post-surface conditioner.^9^ However, the existing literature remains limited regarding the effectiveness of this laser as a surface conditioning regime for GFPs, and hence, further investigation is warranted.

GFP can be cemented with different types of resin cements, which are categorized by polymerization method (light-cured, chemically cured, or dual-cured) and dentin conditioning approach (total-etch or self-adhesive). Light-cured resin cements are infrequently used for the cementation of glass fiber posts because light transmission to the apical region of the root canal is limited.^10^ Consequently, dual-cure resin cements (DCRCs) are considered the material of choice for GFP cementation. Dual-cure systems integrate the advantages of light-activated polymerization with an additional chemical curing mechanism, ensuring adequate polymerization throughout the root canal.^11^ Recent literature has documented limitations with conventional DCRC bonded using the total etch (TE) technique, which requires conditioning with 37% phosphoric acid.^12^ These limitations include technique sensitivity and procedural complexity from multiple steps. To overcome this, a self-adhesive dual-cure resin cement (SADCRC) was introduced to simplify the cementation procedure and reduce the necessary steps.^12^

The current investigation was predicated on the assumption that the R_a_ and PBS of GFP conditioned with H_2_O_2_ and the Nd: YVO_4_ laser would not differ significantly from the control. Furthermore, it was also anticipated that PBS of GFP would not differ significantly when bonded with CDCRC or SADCRC within the same conditioning group. Thus, the objective of the present study was to compare the effects of different post-surface conditioners on Ra and bond strength of GFP bonded using CDCRC and SADCRC.

## METHODOLOGY

The present in vitro study was approved by King Abdulaziz University FC-741-2025; dated June 23, 2025 and followed the in vitro study guidelines CRIS. The study lasted four months, from July 1, 2025 to October 30, 2025. Sixty single-rooted premolars with fully formed apices, free from caries, cracks, root resorption, or fractures, having a straight root canal (curvature ≤ 20°), and no previous root canal treatment. Samples were submerged in 0.2% thymol solution (Caelo, Hilden, Germany). Soft tissues were scraped off using #15 surgical blades (Swann-Morton, Sheffield, England). A double-sided diamond disc (Komet, Santo Andre, SP, Brazil) was used with continuous water irrigation to cut the crown portion and standardize the root length at 15 mm. All samples were embedded in auto polymerizing acrylic resin (Heraeus Kulzer’s Technovit 4000).^8^

### Sample preparation:

Endodontic access cavities were prepared using a high-speed handpiece with a #10 K-file (Dentsply Sirona, Charlotte, NC, USA). Canal patency was confirmed by advancing a #15 K-file until visible at the apical foramen. Working length (WL) was established by shortening the root length by 1 mm. Root canal instrumentation was performed using the ProTaper Universal rotary system (Dentsply Sirona) till F3 finishing file. During preparation, 5-mL of 2.5% sodium hypochlorite (NaOCl) solution (Hedinger, Stuttgart, Germany) was used between each file. After instrumentation, 5 mL of 2.5% NaOCl, 5 mL saline solution, and 5 mL of 17% EDTA (MD Cleanser, Meta Biomed, Cheongwon-gun, Korea) were sequentially added, with EDTA left for one minute as the final disinfectant. The final 10 mL saline wash removed the remaining EDTA. ProTaper Universal paper points (Dentsply Sirona) were used to dry the canals. F3 gutta-percha and AH Plus sealer (Dentsply York, PA, USA) were used in the single-cone technique for obturation. Access cavities were then closed with Cavit (3M ESPE, Maplewood, USA).^13^

### Post space preparation:

The root canal filling was removed to a length of 10 mm using a Peeso reamer (1-5; Mani Inc., Japan). The apical third of the root was left with about 4 mm of gutta-percha, which was confirmed radiographically. The canals were washed with 5 mL of 2.5% NaOCl solution followed by 5 mL of 17% EDTA as a final disinfectant for 1 min. The canals were then dried using absorbent paper.^4^

### Conditioning of GFP:

Eighty-one (White Post DC #2, FGM, Joinville, SC, Brazil) GFP were split into three groups based on the conditioning regime applied (n= 25)

***Group-1:*** No conditioning (Control) GFP was not treated with any conditioner.

***Group-2:*** 24% H_2_O_2-_ Silane. In this group, the GFP (White Post DC #2, FGM, Joinville, SC, Brazil) were immersed in 24% H_2_O_2_ for 60 seconds, then rinsed with water and air-dried using gentle air blasts. Subsequently, a silane coupling agent, Monobond Plus (Ivoclar Vivadent), was applied to the posts using a disposable applicator and left to react for 60 seconds, followed by gentle air-drying of the post surfaces.^14^

***Group-3:* Nd:** YVO_4_ laser- Silane In this group, GFP specimens were surface-conditioned using an Nd: YVO_4_ laser (Frankfurt Laser Corporation, Friedrichsdorf, Germany). The laser was configured to produce surface indentations with a vertical spacing of 200 µm and a depth of 150 µm, following the protocol described by Alkhudhairy et al. Laser irradiation was performed perpendicularly with an 8 ns pulse width, a photo-irradiation speed of 500 mm/s, a frequency of 25 kHz, a wavelength of 1064 nm, and a power density of 5.3 MW/cm^2^. Specimens were exposed for 33 seconds at a working distance of 197 mm above the surface. After laser treatment, the post surfaces were thoroughly cleaned and air-dried.^8^

### R_a_ assessment:

Five posts from each group were subjected to R_a_ analysis using a profilometer (Surfcorder SE700, Kosaka Laboratory, Tokyo, Japan). A stylus (5 µm) on a measuring device was used to scan the sample surface. The scan distance of the stylus was 0.75 mm. For each sample, the R_a_ was measured three times. These three measurements were then averaged to calculate the Ra value.^15^,^16^

***Subgroup-A:* CDCRC** Following 15 seconds of 37% phosphoric acid etching, the root canals were washed and dried with paper points. A microbrush was used to apply All Bond 2 (Bisco Dental, Schaumburg, IL, USA) into the canal. Light curing was performed for ten seconds as per the manufacturer’s instructions. Duo-Link cement was injected into the canals using a syringe tip after preparation. GFP posts were inserted firmly into the canal for 30 s after cement application. Excess cement was removed, and the operator held the post while it was light-cured for 40 seconds.

***Subgroup-B:* SADCRC** Following the manufacturer’s directions, SADCRC (Rely X Unicem, 3M ESPE, St. Paul, MN, USA) was mixed. The cement was injected into the canal and also applied to the posts. Posts were then inserted into the canals using hard finger pressure. The excess cement was removed, and the post was held in position by the operator’s hand while light curing was carried out for 20 secs, in accordance with the manufacturer’s instructions. Samples storage and artificial aging. All specimens were stored in distilled water at 37 °C for one week and then subjected to 10,000 thermocycling cycles between 5 °C and 55 °C, with a dwell time of 30 seconds at each temperature.

### Preparation of samples for analysis of push-out bond strength:

Using a hard tissue microtome (Isomet 1000; Buehler, USA) and constant water irrigation to avoid overheating, sixty samples luted with GFP were transversely sectioned perpendicular to the post beginning at 6 mm from the specimen’s apex. One slice was obtained from each third, i.e., cervical, middle, and apical, measuring 2.0 ± 0.2 mm. One slice from each region was selected at depths of 1, 4, and 7 mm from the coronal to the apical direction.^17^

### PBS testing:

A universal testing machine (EMIC, São José dos Pinhais, SP, Brazil) was used to measure PBS. Each root slice was secured to a metallic device with a central aperture larger than the canal. Metallic plunger (coronal 1.4 mm, middle 1.2 mm, and apical 0.9 mm) was used to apply force to the GFP dentin interface, covering it without contacting the canal walls. Force was applied from apical to coronal at 0.5 mm/min until failure, and values were reported in MPa.^18^

### Failure mode analysis:

Two Investigators used a stereomicroscope (“LYNX” Lawrence & Mayo) at 40× magnification to examine each slice and categorize failure patterns. Fractures are categorized as adhesive, cohesive, and admixed.^6^

### Statistical analysis:

Normality of the data was assessed using the Shapiro-Wilk test.ANOVA and post hoc tests were used to evaluate whether the groups differed significantly. All statistical analyses were conducted using SPSS (version 21.0; IBM Corp., Armonk, NY, USA).

## RESULTS

### Ra assessment:

The Ra of GFP following distinct surface pretreatment techniques is shown in Table-I. The highest Ra (1.32 ± 0.22 µm) was observed in Group-2 (Nd: YVO_4_ laser) samples. Whereas Group-1 (Control) had the lowest Ra (*0.43 ± 0.11* µm). An intergroup comparison revealed that there was no discernible difference between the Group-2(*0.43 ± 0.11* µm) and Group-3 Ra results (p > 0.05).

**Table-I:** Shows the Ra of the glass fiber post following various surface pretreatment methods.

Pretreating agents	Mean ± SD (µm)	p-value!
Group-1: No conditioning (Control)	0.43 ± 0.11 ^b^	< 0.05
Group-2: 24%H_2_O_2_-silane	1.28 ± 0.18 ^a^	
Group-3: Nd: YVO4 laser-silane	1.32 ± 0.22 ^a^	

! ANOVA

(H_2_O_2_): Hydrogen peroxide, (Nd: YVO4) laser: Neodymium-doped yttrium orthovanadate (Nd: YVO4) laser

Same letters in each row show statistically significant difference (p<0.05), Post Hoc Tukey.

### PBS assessment:

Table-II showed the PBS of GFP attached to canal dentin following the use of various adhesive cements and surface pretreatments. The cervical third of Group-3A (Nd: YVO4 laser-Silane + CDCRC) (11.26±1.17 MPa) displayed the highest bond strength outcome. Whereas, the apical third of Group-1A (No conditioning-Silane + CDCRC) (3.78±0.21 MPa) exhibited the lowest bond strength. Intergroup comparison analysis showed that Group-2A (H_2_O_2_-Silane+CDCRC) (Cervical: 11.47±0.52 MPa, Middle: 9.67±0.43 MPa, Apical: 7.02±0.54 MPa) and Group-3A (Nd: YVO4 laser-Silane + CDCRC) (Cervical: 11.26±0.32 MPa, Middle: 9.52±0.54 MPa, Apical: 7.32±0.43 MPa) exhibited comparable scores of bond strength at all three sections (p>0.05). Likewise, Group-2B (H_2_O_2_-Silane + SADCRC) (Cervical: 9.41±0.27 MPa, Middle: 10.80±0.19 MPa, Apical: 11.31±0.21 MPa) and Group-3B (Nd: YVO4 laser-Silane+SADCRC) (Cervical: 9.56±0.17 MPa, Middle: 10.76±0.23 MPa, Apical: 11.23±0.17 MPa) also showed no discernible variation in their PBS results at all three thirds of the canal(p>0.05).

**Table-II:** PBS of GFP bonded to the canal dentin after applying different adhesive cements and surface treatments.

Pretreating agents	Mean ±SD Cervical (MPa)	Mean ± SD Middle (MPa)	Mean ± SD Apical (MPa)	P-value!
Group-1A: No conditioning -Silane + CDCRC	6.21±0.91 ^c, A^	5.09±0.13 ^c, B^	3.78±0.21 ^c, C^	*< 0.05*
Group-1B: No conditioning -Silane + SADCRC	5.12±0.20 ^d, A^	5.86±0.11 ^d, B^	6.75±0.21 ^d, C^	
*Group-2A: 24% H_2_O_2_* -*Silane + CDCRC*	11.47±0.52 ^b, A^	9.67±0.43 ^b,B^	7.02±0.54 ^b, C^	
*Group-2B: 24% H_2_O_2_* -*Silane* *+ SADCRC*	9.41±0.27 ^a, A^	10.80±0.19 ^a,B^	11.31±0.21 ^a, C^	
*Group-3A: Nd: YVO4 laser-Silane* + *CDCRC*	11.26±0.17 ^b, A^	9.52±0.54 ^b, B^	7.32±0.43 ^b, C^	
Group-3B: Nd: YVO4 laser-Silane + SADCRC	9.56±0.17 ^a, A^	10.76±0.23 ^a,B^	11.23±0.17 ^a, C^	

! ANOVA

(H_2_O_2_): Hydrogen peroxide, (Nd: YVO4) laser: Neodymium-doped yttrium orthovanadate (Nd: YVO4) laser

Data with Different superscript lower-case alphabets denote statistically significant differences within the same column (p < 0.05). Data with different upper-case letters denote significant differences within each row (p < 0.05), Post Hoc Tukey.

However, Group-1A (No conditioning-Silane+CDCRC) (Cervical: 6.21±0.91 MPa, Middle: 5.09±0.13 MPa, Apical: 3.78±0.21 MPa) and Group-1B (No conditioning-Silane + SADCRC) (Cervical: 5.12±0.20 MPa, Middle: 5.86±0.11 MPa, Apical: 6.75±0.21 MPa) displayed a significant difference in the PBS outcomes(p < 0.05). Regarding intragroup comparisons, bond strength in the CDCRC groups declines from the cervical to the apical third (p < 0.05). In contrast, in the SADCRC groups, PBS increases from the cervical to the apical third (p < 0.05).

### Failure mode analysis:

**Fig.1:**
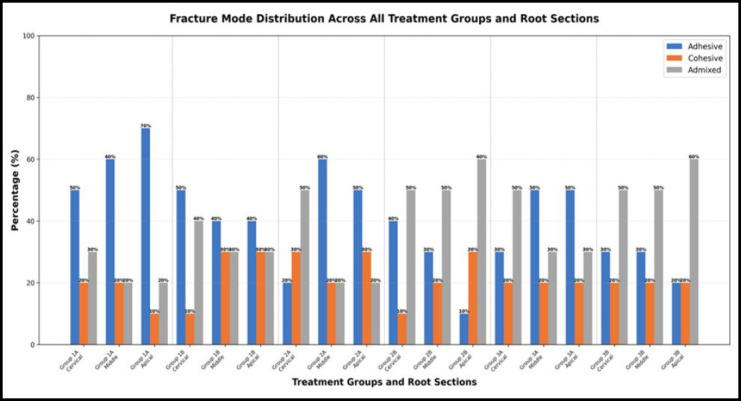
Distribution of Adhesive, Cohesive, and Admixed Failure Modes Across All Groups.

Fig.1 presents the fracture mode analysis across the tested groups. It was observed that admixed failures were more common in all three sections of H2O_2_ + SADCRC and Nd: YVO4 laser + SADCRC. Whereas the cervical section of H_2_O_2_ + CDCRC and Nd: YVO4 laser + CDCRC displayed admixed failures. On the other hand, the middle and apical sections presented adhesive failures. No conditioning-Silane+CDCRC and No conditioning -Silane + SADCRC also displayed adhesive failures the most at all three thirds.

## DISCUSSION

The current investigation examined R_a_ and PBS of GFP conditioned with H_2_O_2_-silane, and the Nd:YVO_4_ laser did not differ significantly from the control. Furthermore, it was also anticipated that PBS of GFP would not differ significantly when bonded with CDCRC or SADCRC within the same conditioning group. Based on the findings, both H_2_O_2_-silane- and Nd: YVO_4_-laser-treated samples showed significantly different R_a_ and bond strength compared to the control, thereby rejecting the primary null hypothesis. Additionally, significant differences in bond strength were observed between the SADCRC and CDCRC groups, thereby rejecting the secondary hypothesis. The PBS test was employed because it is the most clinically relevant method, as it applies a load along the longitudinal interface between the adhesive material and dentin.^19^

The present study found that GFP treated with an Nd: YVO_4_ laser and H_2_O_2_ pretreatment displayed comparable Ra scores, both of which were significantly lower than the control. PBS among these groups at all three sections was comparable when bonded with the same luting cement. The effectiveness of Nd: YVO_4_ laser as a surface conditioning method aligns with previous research by AlRefeai et al., who reported that laser irradiation increases surface roughness by creating controlled micro-irregularities. These surface alterations significantly increase the bonding area and enhance micromechanical retention between the fiber post and resin cement.^9^ Nd: YVO_4_ laser treatment, attributed to partial resin matrix removal through photomechanical, photochemical, and photothermal interactions.^9^ Similarly, H_2_O_2_ conditioning produced acceptable Ra and BS outcomes. As an oxidizing agent, H_2_O_2_ selectively degrades the epoxy resin matrix through electrophilic reactions, creating surface irregularities that enhance micromechanical interlocking.^20^

Bond strength in CDCRC groups declined from the cervical to the apical third (p<0.05), whereas SADCRC groups showed increased PBS from the cervical to the apical third. CDCRC bonding relies on micromechanical retention through smear layer removal and dentinal tubule exposure.^21^ The 37% phosphoric acid etching efficiently eliminates the smear layer, particularly in the cervical region.^22^ Reduced apical bond strength may result from a smaller diameter and decreased density of dentinal tubules in the apical third, along with debris contamination and reduced light penetration.^23^ Conversely, SADCRC groups demonstrated increased PBS from cervical to apical regions, potentially linked to reduced tubule density, favoring bond strength and higher moisture tolerance. However, cervical third bond strength was reduced compared to the control due to limited demineralization capacity. The cement’s low pH fails to adequately demineralize dentinal tubules, preventing sufficient infiltration of methacrylate phosphate ester and creating interfacial gaps.^23^,^24^

Regarding fracture modes, admixed failures predominated in H_2_O_2_+SADCRC and Nd: YVO_4_ laser + SADCRC groups across all sections. Coronal sections of H_2_O_2_+CDCRC and Nd: YVO_4_ laser + CDCRC also displayed admixed failures, while middle and apical sections presented adhesive failures. The conditioning groups predominantly exhibited adhesive failures, consistent with the bond-strength results.^25^

### Novelty and Contribution to Medical Literature:

This is the first study demonstrating Nd: YVO_4_ laser as a superior, safer alternative to hydrogen peroxide for fiber post surface treatment—addressing a critical gap where current chemical conditioning protocols pose oxidative degradation risks, require prolonged application times, and show inconsistent clinical outcomes. The pioneering evaluation of laser-silane synergy establishes that combining photothermal micro-ablation with chemical coupling agents achieves bond strengths exceeding 11 MPa—well above the 5 MPa clinical threshold for withstanding masticatory forces—while eliminating post-operative sensitivity and cytotoxicity concerns associated with H_2_O_2_ residues.

### Limitations:

It includes the in vitro design, which does not fully reflect the complexity of the oral environment; use of a single laser parameter set, which may have influenced outcomes; unstandardized cement layer thickness, which may have affected stress distribution; and aging limited to thermocycling without mechanical loading.

## CONCLUSIONS

A neodymium-doped yttrium orthovanadate laser can be a suitable alternative to hydrogen peroxide. Self-adhesive resin cement exhibited satisfactory performance in all three thirds of the canal.

### Recommendations:

Future investigations should employ atomic force microscopy for deeper surface analysis. Additional in vivo and laboratory research is required to validate these findings.
